# Recurrent Glioblastoma Treatment: State of the Art and Future Perspectives in the Precision Medicine Era

**DOI:** 10.3390/biomedicines10081927

**Published:** 2022-08-09

**Authors:** Augusto Leone, Antonio Colamaria, Nicola Pio Fochi, Matteo Sacco, Matteo Landriscina, Giovanni Parbonetti, Matteo de Notaris, Giulia Coppola, Elena De Santis, Guido Giordano, Francesco Carbone

**Affiliations:** 1 Department of Neurosurgery, Städtisches Klinikum Karlsruhe, 76133 Karlsruhe, Germany; 2 Department of Neurosurgery, Charité Universitätsmedizin Berlin, 10117 Berlin, Germany; 3 Department of Neurosurgery, Riuniti Hospital, 71122 Foggia, Italy; 4 Department of Neurosurgery, University of Foggia, 71122 Foggia, Italy; 5 Unit of Medical Oncology and Biomolecular Therapy, Department of Medical and Surgical Sciences, University of Foggia, 71122 Foggia, Italy; 6 Department of Neurosurgery, “Rummo” Hospital, 82100 Benevento, Italy; 7 Department of Radiological, Oncological and Pathological Sciences, Sapienza University of Rome, 00185 Roma, Italy; 8 Department of Anatomical Histological Forensic Medicine and Orthopedic Sciences, Sapienza University of Rome, 00185 Roma, Italy

**Keywords:** recurrent glioblastoma, brain tumor, review, glioblastoma treatment, chemotherapy, regorafenib, target therapy, immunotherapy, molecular profile, clinical trial

## Abstract

Current treatment guidelines for the management of recurrent glioblastoma (rGBM) are far from definitive, and the prognosis remains dismal. Despite recent advancements in the pharmacological and surgical fields, numerous doubts persist concerning the optimal strategy that clinicians should adopt for patients who fail the first lines of treatment and present signs of progressive disease. With most recurrences being located within the margins of the previously resected lesion, a comprehensive molecular and genetic profiling of rGBM revealed substantial differences compared with newly diagnosed disease. In the present comprehensive review, we sought to examine the current treatment guidelines and the new perspectives that polarize the field of neuro-oncology, strictly focusing on progressive disease. For this purpose, updated PRISMA guidelines were followed to search for pivotal studies and clinical trials published in the last five years. A total of 125 articles discussing locoregional management, radiotherapy, chemotherapy, and immunotherapy strategies were included in our analysis, and salient findings were critically summarized. In addition, an in-depth description of the molecular profile of rGBM and its distinctive characteristics is provided. Finally, we integrate the above-mentioned evidence with the current guidelines published by international societies, including AANS/CNS, EANO, AIOM, and NCCN.

## 1. Introduction

**Figure 1:**
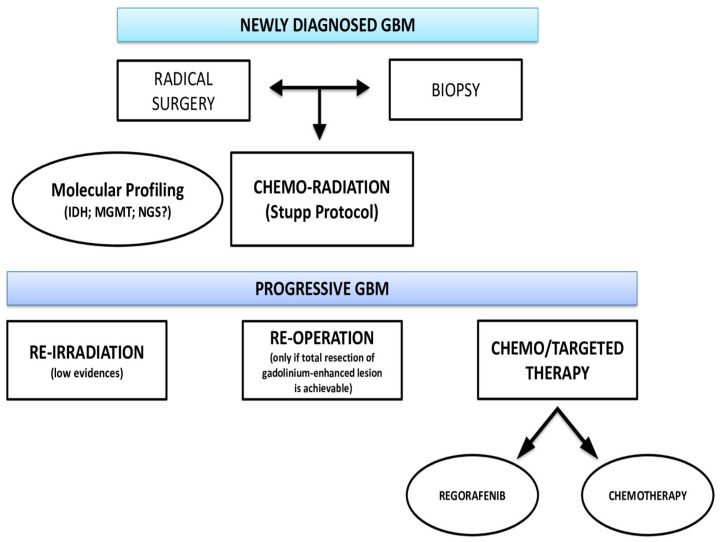
Current diagnostic and therapeutic management for rGBM.

Glioblastoma (GBM) represents the most common, yet deadly, brain tumor in the adult population. Despite novel surgical and pharmacological treatments, its prognosis remains dismal, with the median survival not exceeding 14 months and the 5-year mortality rate being 97% [1]. Currently, the standard of care (SOC) for newly diagnosed patients (Stupp’s protocol) comprises gross total surgical resection (GTR) followed by radiation therapy (RT) plus concomitant chemotherapy (CT) with temozolomide (TMZ) for six weeks and adjuvant chemotherapy of six cycles with an alkylating agent [2]. Nevertheless, an analysis of patients treated by such a protocol showed no significant reduction in the recurrence rates between individuals treated with RT alone and RT plus concomitant and adjuvant CT, suggesting that combination therapy, although effectively reducing tumor aggressiveness at the initial stage, does not significantly alter the disease course [3]. Therefore, the management of recurrent GBM (rGBM) continues to challenge neurosurgeons and neuro-oncologists, since no standard treatment has yet been validated, and solely empirical indications exist (Figure 1).

The recurrence of high-grade gliomas is nearly ubiquitous [4], with most recurrences presenting within 2 cm of the initial tumor margin [5] and approximately one-third of rGBMs resurging in the contralateral hemisphere, a different lobe, and rarely infratentorially [6]. Attempts to compare the genomic and molecular profiles of primary GBM (pGBM) with rGBM have largely demonstrated inconsistencies and significant disparities between the two tumors that therefore appear as separate molecular entities [7]. This confounding factor could be the reason why targeted molecular therapies have partially failed to achieve auspicated results, such as those obtained for non-small-cell lung cancer and colorectal cancer [8,9]. Notwithstanding the urgent need to investigate rGBM’s molecular profile to elucidate its unique features, current studies mostly focus on pGBM, possibly due to the scarce tissue accessibility and availability of rGBM. As a matter of fact, only 30% of recurrences are surgically treated, and most patients succumb during the first cycle of adjuvant chemotherapy due to an undiagnosed recurrence [10,11]. Therefore, let alone the difficulties associated with the immunological niche that characterizes the central nervous system that ultimately limits the distribution of conventional systemic drugs, several factors depending on the distinctive cell-intrinsic and microenvironmental features of neural tissue hinder optimal drug development for primary and progressive brain tumors. Notably, specific drug-resistance mechanisms further limit the efficacy of novel therapeutical approaches and render these malignancies unique challenges that, together with their relative rarity, have attracted a small and fragmented research community.

In the present review, we outline and compare different molecular targets selectively inhibiting oncogenic pathways and regulating the tumor microenvironment. Moreover, we sought to critically summarize the advancements in the management of rGBM achieved in the last 5-year period with the use of novel targeted therapeutic agents and immunotherapy, and analyze data gathered from ongoing clinical trials. Lastly, we outline future perspectives and technological advancements, such as oncolytic viruses and dendritic cell vaccines, that could optimize drug absorption and efficacy, consequently improving the overall survival (OS) and progression-free survival (PFS) of patients affected by rGBM.

## 2. Materials and Methods

**Figure 2:**
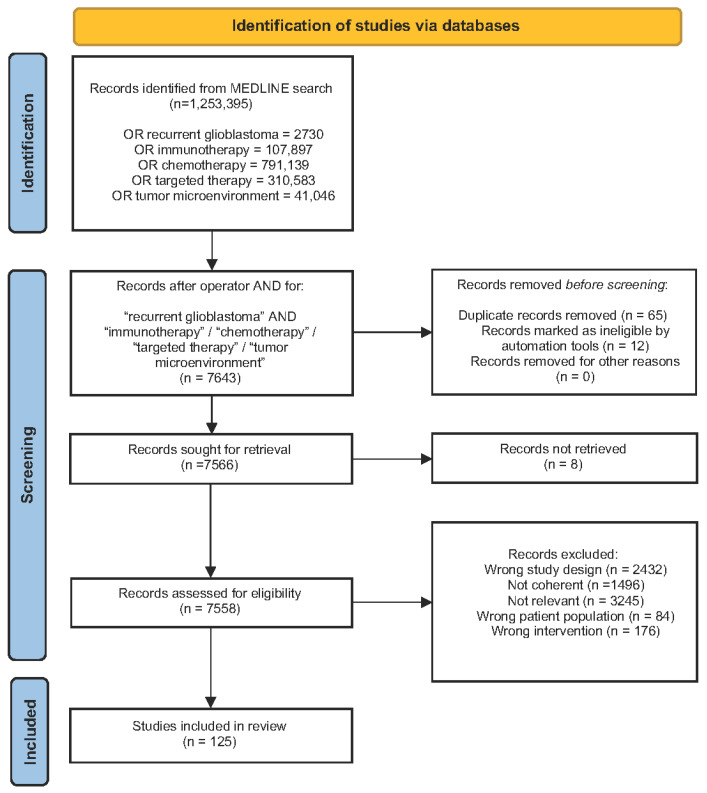
Preferred Reporting Items for Systematic Reviews and Meta-Analyses protocol used for the present review.

A qualitative review of the literature was performed in compliance with the updated Preferred Reporting Items for Systematic Reviews and Meta-Analyses (PRISMA) 2020 guidelines [12]. The screening was performed by reviewing manuscripts published in the last 5 years up to March 2022 using the electronic database MEDLINE/PubMed. The primary search terms included “recurrent glioblastoma”, “immunotherapy”, “chemotherapy”, and “targeted therapy” in the article titles, abstracts, and keywords in various combinations. The extracted citations were then checked for duplicates. Any irrelevant research, review articles, meeting abstracts/summaries, and editorials not meeting the scope of the present investigation were excluded. A total of 7558 articles met the eligibility criteria for our qualitative review, and 7433 were excluded through an automated system (Covidence) [13]. Publications solely addressing pGBM patients’ management and outcomes, as well as non-human studies, were considered as “wrong study population”, whereas “wrong study design” was defined as case reports, series, or editorials describing cases of progressive disease lacking standardized data regarding rGBM treatment and outcomes. All records without a clear conclusions section and sound results were excluded as “not coherent”. Publications mentioning non-conventional or complementary/alternative therapeutical approaches were considered as “wrong intervention”. Finally, one hundred and twenty-five papers were included in the qualitative analysis (Figure 2). The citations of the examined manuscripts were also screened for this review. Notably, publications discussing nanomedicines were not considered in this study, since most of the published reports in this regard focused on primary GBM and gliomas in general, which are not the main targets of the present article. Currently, phase-Ib/II clinical trials testing nanomedicines for the treatment of rGBM are still under evaluation with data that need to be analyzed or not yet closed. In addition, the ClinicalTrials.gov website (accessed on 5 May 2022) was analyzed, and “recruiting,” “active, not recruiting,” and “not yet recruiting” trials in rGBM were selected (Table 1). The National Cancer Institute Drug Dictionary was consulted to verify the mechanism of action of screened drugs (Table 2). Trials with negative or not clinically relevant results were excluded from this article. Furthermore, European Association of Neuro-Oncology (EANO), European Society of Medical Oncology (ESMO), American Association of Neurological Surgeons (AANS)/Congress of Neurological Surgeons (CNS), American Society of Clinical Oncology (ASCO), Associazione Italiana di Oncologia Medica (AIOM), and National Comprehensive Cancer Network (NCCN) abstracts published during the last five years were evaluated in order to obtain the most recent clinical data regarding drugs adopted for the management of rGBM. ESMO and ASCO guidelines were not included in the present review, since the last published ESCO guideline examining rGBM dates to 2014, and no relevant ASCO abstracts were published on the matter in the last five years.

## 3. Locoregional Treatment of rGBM

Less than 50% of newly diagnosed patients with pGBM are deemed eligible for radical or cytoreductive surgery due to tumor inoperability or poor candidacy for surgery [14]. These numbers drop even lower for patients presenting with rGBM, where reintervention rates do not exceed 30%, with some studies reporting the ability to perform a second surgery in less than 10% of patients [15,16]. In addition, even when feasible, the impact of repeat surgery on prognosis remains controversial [17]. In their comprehensive review, Robin et al. [18] examined previously reported findings for a total of 2717 patients undergoing a second or third surgery for rGBM. Among the 33 studies considered, only 20 saw a role for reoperation in the case of progressive GBM, whereas ten studies saw either no benefit from reoperation or adopted alternative treatment strategies, including radiation therapy and chemotherapy. Finally, amid the abovementioned publications that contemplated repeat surgery for rGBM, thirteen articles with the addition of three other reports deemed the extent of resection of both initial and second surgery to be of prognostic value. Despite there being less available evidence for rGBM when compared with pGBM, GTR is estimated to provide a similar survival benefit of 3–5 months in both cases [19]. This could be associated with the generally accepted knowledge that less residual tumor corresponds to longer PFS; nonetheless, it should be considered that patients elected for reoperation are relatively fitter and present higher Karnofsky scores (KPS) when compared with non-eligible patients [19]. Furthermore, reoperation for rGBM has been associated with higher complication rates for morbidity (13–69%) and mortality (0–11%), precluding further treatment options, such as systemic chemotherapy, due to insufficient postoperative performance status [15,18,20]. As a consequence, more attention is given to minimally invasive salvage techniques, such as stereotactic radiotherapy (SRT), which represents a viable treatment option, especially in patients with long time-to-recurrence. Recently, Yaprak et al. [21] showed the benefits of SRT in a cohort of 42 patients diagnosed with rGBM following a first-line treatment consisting of resective surgery plus adjuvant TMZ and radiation therapy, who underwent salvage stereotactic treatment. With a median of three fractions at a prescription dose of 20 Gy (range, 18–30), the group was able to demonstrate a statistically significant difference in the survival time between the SRT-treated population (mean, 30 months; range, 9–123) and the patients who could not receive SRT (mean, 14 months; range, 1–111) (*p* = 0.001). Despite growing evidence and promising results, locoregional therapy for rGBM does not yield the desired benefits, also since no targeted surgical or radiotherapy protocols have been proposed based on the biological features hosted by this malignancy so far.

## 4. Molecular Footprints of rGBM

Similar to the advancements witnessed in the fields of oncology, immunology, and dermatology, neuro-oncological treatments have recently shifted from unspecific protocols based on cytotoxic systemic drugs toward a more precise and patient-specific approach. The search for targeted and safer therapies has heightened the urgent need for preclinical and clinical studies on pGBM and progressive GBM models and patients to gather practical insights that could lead to better drug engineering and assure multiple pharmacological options for different molecular subtypes.

As highlighted by the 2021 WHO classification of brain tumors, the classification paradigm once based solely on histological, immunohistochemical, and radiological appearance characterizing the lesion now integrates previous models with molecular tumor profiling technologies for the recognition of its distinctive and patient-specific footprints, which appear to be better markers of prognostic and therapeutical value [22].

In the following section, a summary of the peculiar molecular trademarks of rGBM is presented, along with a description of the latest pharmacological advancements, taking advantage of recent knowledge gained from preclinical and clinical studies.

### 4.1. O-6Methylguanine-DNA Methyltransferase

Although the DNA repair protein O-6Methylguanine-DNA methyltransferase (MGMT) gene is ubiquitously expressed throughout most human tissues, the regulation of protein production varies greatly based on the degree of epigenetic silencing through gene promoter methylation. Given the ability of MGMT to encode a DNA-repair protein that is established to reduce the therapeutic effects of alkylating agents (i.e., TMZ), its methylation and, therefore, its suppression have long been considered a positive predictive factor of treatment response and OS in patients diagnosed with pGBM [23,24,25,26]. Furthermore, the silencing or downregulation of MGMT, occurring in 45% of GBMs, appears to correlate with post-recurrence survival, with MGMT-promoter methylated patients showing longer survival times than their unmethylated counterparts (mean, 3–4 months) [27,28,29]. Given the high genetic variability that exists between pGBM and rGBM, it is worth mentioning that the epigenetic silencing of MGMT is preserved through tumor progression in 70–90% of cases, thereby serving as a relatively stable prognostic marker [30,31]. In their meta-analysis of clinical trials, Binabaj et al. [26] reported a significant correlation between OS and MGMT promoter methylation assessed with univariate analysis (*p* = 0.001), although PFS was not observed to be related to MGMT silencing in the 10 studies explored. On the other hand, Cantero et al. [32] showed that MGMT methylation, along with isocitrate dehydrogenase (IDH) mutation, is not detected in a notable proportion of long-term survivors examined by next-generation sequencing, leaving concerns over its prognostic value.

Notwithstanding the controversial prognostic significance, MGMT remains an attractive molecular marker for systemic chemotherapy for both pGBM and rGBM. As a matter of fact, TMZ, an alkylating agent capable of adding alkyl groups to guanines, represents the current standard chemotherapy treatment for newly diagnosed GBM, and it is administered as a concomitant therapy to surgical resection followed by adjuvant TMZ maintenance for six cycles, showing significantly better results in MGMT-methylated populations across various studies [33,34,35]. Recent evidence also advocates for MGMT promoter methylation as a predictive biomarker reflecting treatment response to alkylating agents in progressive disease. For instance, the AVAREG trial, assessing fotemustine and bevacizumab, and the BELOB trial, assessing the role of single-agent bevacizumab/lomustine or a combination of the two in patients with rGBM, confirmed the predictive value of MGMT silencing in the estimation of OS in this population [36,37,38].

For patients that do not present MGMT promoter methylation, however, new ways to improve response rates are currently under review. A phase-II trial investigating the role of O(6)-benzylguanine in adults with progressive, TMZ-resistant gliomas reported favorable results for anaplastic glioma, but was rather inconclusive in the case of rGBM, notwithstanding the relatively high hematopoietic toxicity of this combination, therefore prompting further research for the assessment of the real-life role of this irreversible inhibitor of the DNA repair protein coded by the MGMT gene [38]. In a recent study, Yamada et al. [39] demonstrated the time- and dose-dependent role of riluzole, a metabotropic glutamate receptor 1 inhibitor, in slowing the growth of human GBM cell lines. Moreover, they showed the independent ability of riluzole to suppress MGMT expression in MGMT methylated cells and TMZ-induced MGMT upregulation (*p* < 0.01).

### 4.2. Vascular Endothelial Growth Factor

Another distinctive molecular feature of rGBM is represented by the overexpression of vascular endothelial growth factor (VEGF). Through the activation of peculiar molecular pathways, this protein plays a crucial role in regulating complex biochemical mechanisms, including the proliferation, migration, and differentiation of vascular endothelial cells following hypoxic stress [38,39,40,41]. Its overexpression is considered pivotal in GBM progression, and the inhibition of various components of the angiogenetic axis has, therefore, been extensively investigated in various clinical trials. Bevacizumab, a VEGF-A targeting monoclonal antibodies, was the first drug to be approved for the treatment of newly diagnosed and progressive GBM for its ability to downregulate VEGF expression [36]. Although bevacizumab alone did not significantly improve OS in rGBM when compared with lomustine [28], various trials have investigated the possibility of adding cetuximab, tandutinib, and sorafenib, a chimeric antibody targeting the epidermal growth factor receptor (EGFR), a platelet-derived growth factor receptor (PDGFR), and VEGF receptor (R), respectively, to improve survival rates and PFS. Unfortunately, these phase-II trials failed to show statistical significance, since these combinations achieved similar results to bevacizumab alone [42,43,44]. D’alessandris et al. recently conducted a triple-armed, prospective cohort study investigating the administration of bevacizumab alone or in combination with erlotinib or sirolimus, taking into consideration the tissutal expression of VEGF, epidermal growth factor receptor variant III (EGFRvIII), and phosphatase and tensin homolog (PTEN) in patients presenting with rGBM [45]. They were able to demonstrate higher clinical benefits (mean, 71% of patients) than those achieved in the EORTC 2016 trial in terms of PFS at 6 and 12 months [28]. These results showed that a personalized therapy tailored to the molecular and genetic profile of rGBM could sensibly improve patients’ outcomes and should, therefore, become SOC after the foreseen randomized controlled trials (RCTs).

### 4.3. Epidermal Growth Factor Receptor

EGFR belongs to the family of erythroblastic oncogene B (ErbB) transmembrane tyrosine kinase receptors and is known to regulate a complex signaling cascade driving cell proliferation, differentiation, division, and survival [43,44,45,46,47,48]. Its role in oncogenesis and progression has been confirmed in various types of solid tumors, and its pharmacological regulation is currently SOC for several of these malignancies [49,50]. Since the first description of gene amplification and overexpression in human GBM in 1985 [51], EGFR structure and regulating function, along with its most-frequently mutated form (EGFRvIII), have been the focus of both pre- and clinical trials exploring different drug generations [52,53]. Nonetheless, despite gene amplification being reported in more than half of GBMs [54], promising early results have failed to keep up with expectations. As a matter of fact, first-generation EGFR inhibitors (erlotinib and lapatinib) that compete with ATP, thus blocking the activation of the receptor, as well as second-generation drugs, including afatinib and dacomitinib, that irreversibly bind the tyrosine kinase domain demonstrated only limited efficacy in phase-II clinical trials [55,56,57,58]. Future directions involve third-generation irreversible EGFR inhibitors, such as osimertinib and rociletinib, which are currently under investigation in phase-II RCT in EGFR-activated rGBM (NCT03732352). Interesting results were shown by the INTELLANCE-2/EORTC 1410 study [59]. In this randomized phase-II study, the role of depatuxizumab mafodotin (Depatux-M), an antibody conjugated with toxin monomethylauristatin-F that inhibits microtubule polymerization in EGFR-amplified rGBMs was investigated as the sole agent or in combination with TMZ versus SOC with Lomustine or TMZ in progressive EGFR-positive GBM. Although the primary endpoint of efficacy was not achieved at a 15-month follow-up, the combined administration showed a positive trend with regard to survival at longer follow-up times (28.7 months), with a statistically significant difference in OS between the two arms (hazard ratio, 0.66) and corneal epitheliopathy being reported as the most common adverse effect (25% of cases).

### 4.4. Telomerase Reverse Transcriptase

Mutations of the telomerase reverse transcriptase (TERT) promoter show the highest retention rates from pGBM to rGBM (~90%) among several genetic abnormalities investigated to date [27,60]. Encoding a catalytic subunit of the enzyme telomerase, TERT controls a rate-limiting de novo addition of telomere repeats at chromosomal ends, serving as a prognostic factor for pGBM and progressive disease [61]. As a matter of fact, TERT promoter mutation, in combination with IDH wild-type status, has been demonstrated to correlate with poor OS in patients with primary and rGBM [61,62,63]. Despite rGBM frequently hosting TERT promoter mutations supporting tumor cells’ immortalization, the pharmacological inhibition of TERT abnormal transcription has not yet been considered for extensive investigation, let alone sparse preclinical studies on GBM models [64,65,66].

### 4.5. Platelet-Derived Growth Factor Receptor

Platelet-derived growth factor receptors (PDGFRs) are tyrosine kinase receptors located on the cell’s surface, exploiting their regulatory role in cell proliferation, cellular differentiation, cell growth, development, and promoting tumor growth through autocrine stimulation [67]. For more than twenty years, the hyperexpression of PDGFRɑ (one of the four types of receptors belonging to this family) has been considered an initiating event in the development of gliomas, particularly in high-grade tumors [68]. To elucidate a possible therapeutic benefit derived from the inhibition of PDGFR-mediated activity, several RCTs have been conducted exploring imatinib, a tyrosine kinase inhibitor, alone or in combination with hydroxyurea [69,70,71]. However, apart from a few promising therapeutic results, cases of intratumoral hemorrhage, possibly due to iatrogenic pericyte recruitment, as well as the lack of sound efficacy, suggest that new ways to benefit from PDGFR-dependent metabolic processes regulation are warranted.

### 4.6. Regorafenib, a Multi-Targeted Tyrosine Kinase Inhibitor

Regorafenib is an oral multikinase receptor inhibitor regulating different molecular pathways, including tyrosine kinase receptor with immunoglobulin and EGF homology domain 2 (TIE2), proto-oncogene receptor tyrosine kinase (KIT), rearranged during transfection gene (RET), VEGFR1–3, PDGFR, proto-oncogene, serine/threonine kinase 1 (RAF1), fibroblast growth factor receptor 3 (FGFR), and proto-oncogene, serine/threonine kinase (BRAF). It is currently a viable option as a monotherapy for the treatment of gastrointestinal stromal tumors, colorectal cancer, and hepatocellular carcinoma [72,73,74]. Since its first preclinical assessment by Wilhelm et al. [75] in 2011, the administration of regorafenib in animal models and patients with rGBM has been evaluated either as a single therapy or in combination with other agents, such as lapatinib, sorafenib, and lomustine [76,77,78]. In line with the previously reported promising results, Lombardi et al. [79] aimed to investigate the efficacy and safety of regorafenib, comparing it against lomustine in patients with documented disease progression after surgical resection followed by radiotherapy and TMZ chemoradiotherapy. Their randomized, multicentric, open-label, phase-2 trial demonstrated a statistically significantly longer OS in patients treated with regorafenib, with similar results with regard to PFS and disease control. Nonetheless, the difference in quality of life did not show statistical significance between the two arms. It is worth mentioning that these results refer to patients with higher KPS, as usually seen in clinical practice, since the trial only enrolled subjects with performance status scores ≥ 70. After the REGOMA trial, the National Coalition for Cancer Survivorship (NCCS) 2021 guidelines followed the AIOM and included regorafenib as the first line of pharmacological treatment in patients with progressive disease [80,81]. Recently, a large monocentric, real-life retrospective study further reported promising results with the administration of regorafenib as monotherapy, with grade-3 drug-related adverse events occurring in 18% of patients, and one patient (2%) reporting a grade-4 adverse event (maculopapular rash) [82].

## 5. A Promising Future Direction: Immunotherapy

Amidst the experimental strategies currently under investigation for rGBM, a major role lies within the field of immunotherapy, a novel, widely adopted approach in different specialties, based on the ability to engineer host immune cells to recognize and destroy cancer cells, either by passive immunotherapy (antibodies and immune cells) or active immunotherapy (cancer vaccines) [83,84]. The recently discovered lymphatic drainage system, along with the dural venous sinuses in the central nervous system, have spurred further hopes regarding the distribution and efficacy of systemically administered drugs, overcoming one of the most crucial obstacles yet: penetration of the blood–brain barrier (BBB) [85,86,87,88]. Additionally, drug resistance to conventional therapies exhibited by progressive GBM further explains why immunotherapy represents the leading future perspective, as testified by the numerous RCTs currently underway. Although a wide variety of immunotherapy strategies, including cancer vaccines and oncolytic viruses, are now under evaluation, adoptive T-cell therapies and immune checkpoint inhibitors have achieved the most robust results to date [88]. Notwithstanding the promising benefits that this pioneering pathway could grant, it is worth mentioning that the implementation of immunotherapy for rGBM is inevitably grounded in our understanding of its molecular biomarkers, both for a proper patient selection, as well as a clearer comprehension of tumor progression [87].

In the following sections, an overview of the current state of the art of the most relevant immunotherapeutic agents is presented, along with the results of the recently conducted, as well as still undergoing, clinical trials.

### 5.1. Adoptive T-Cell Therapy

Adoptive T-cell therapies can be divided into tumor-infiltrating lymphocytes (TILs), T-cell receptors (TCR), and chimeric antigen receptor T (CAR-T) cells [89]. With the help of engineered T-cells previously isolated from tumor specimens, this treatment aims to elicit a durable response against tumor-specific antigens. Notwithstanding the promising effects on cancer growth inhibition following direct administration, TILs and TCR only demonstrated vigorous antineoplastic activity against melanomas [89,90]. As a matter of fact, marginal progress was reported in TIL clinical trials for gliomas, whereas none have hitherto been initiated for TCR. Not only does the intrinsic necessity of TILs for an immunogenic and accessible tumor site preclude an effective impact on cancer cells, but the major histocompatibility complex (MHC) restriction also seems to represent the main obstacle for these therapies to be considered in glioblastoma treatment [89]. Conversely, interesting results were shown for CAR-T cells, whose main advantage is represented by the ability to bypass the MHC antigen presentation mechanisms, as well as the needlessness of co-stimulatory signals for activation [89,90].

Already approved for B cell lymphomas and leukemia, CAR-T cell therapy is at the forefront of the adoptive T-cell category for rGBM. It consists of autologous or allogeneic T-cells engineered to identify specific tumor antigens. The cells are then administered back to the patient, inducing substantial antitumor immune responses [91,92]. The main targets of these cells in GBM include EGFRvIII, interleukin-13 receptor subunit alpha-2 (IL-13Ra2), and human epidermal growth factor receptor 2 (HER2), which are already under evaluation in several clinical trials with promising results, as shown by the median survival time of 11.1 months from T-cell infusion and 24.5 months from reported diagnosis with the use of some agents [93,94,95,96,97,98].

However, concerns remain regarding the safety of CAR-T therapies, since elevated intracranial pressure and associated encephalopathy are commonly observed adverse effects in B-cell lymphoma patients, therefore raising concerns regarding their safety and suggesting the necessity for further extensive investigation [99,100]. Additionally, it is worth mentioning that single-antigen CAR-T cell therapies could eventually lead to antigen escape in tumor cells, a common feature in GBM, hence suggesting the necessity for a multiple-antigen therapy. A trivalent CAR-T cell therapy, directed against HER2, IL-13Ra2, and ephrin type-A receptor 2 (EphA2), demonstrated increased antitumor activity when compared with bivalent and single CAR-T cell therapy in a murine model [101]. In conclusion, apart from clinical complications, it is noteworthy that the hefty prices of such therapies may prohibit their widespread adoption [102].

### 5.2. Immune Checkpoint Inhibitors

Given the distinctive upregulation of immune checkpoint receptors in gliomas, the adoption of checkpoint inhibitors could represent a relatively unusual strategy in immunotherapy. Rather than direct activation of the immune system, this therapeutic approach consists of coinhibitory agents targeting both T-cell-mediated and inflammatory responses [85,89]. Molecules under evaluation include cytotoxic T-lymphocyte antigen 4 (CTLA-4), programmed cell death protein 1 (PD-1), its ligand PD-L1, T-cell immunoglobulin, and mucin domain 3 (TIM-3) [89,103]. The former has shown encouraging results in murine glioma models, demonstrating a 75% rate of tumor regression when combined with PD-1, regardless of tumor advancement [104]. Despite direct clinical beneficial evidence concerning CTLA-4 inhibitors for the treatment of rGBM, a variety of clinical trials are currently ongoing, with these agents remaining unavailable for commercial use [105]. However, several concerns have been raised about concomitant steroid use, generally administered to reduce peritumoral edema, due to its ability to substantially weaken the effects of checkpoint inhibition [106,107]. Additionally, the sole administration of checkpoint inhibitors in monotherapy regimens has been shown to be associated with serious, even fatal, side effects, which appeared to be almost nonexistent when administered in combination with other immunotherapies, which are already under evaluation [108]. Notwithstanding a series of clinical trials using combinatorial checkpoint blockade, which failed to deliver the expected results, it is worth mentioning that Pembrolizumab, a PD-1 antibody, showed a significant increase in OS during a single-arm phase-II clinical trial, involving 35 surgically resectable rGBM patients. In addition to a considerable increase in survival (417 days vs. 228.5 days in the adjuvant group), PFS also showed positive results, with 99.5 days in patients treated with Pembrolizumab against 72.5 days in the adjuvant group [109].

### 5.3. Peptide Vaccines

With a length of about 8–30 amino acids, peptide vaccines can target either tumor-specific antigens (TSA), exclusively expressed by malignant cells, or tumor-associated antigens (TAA), which are ubiquitary [110]. After recognizing these specific neoantigens, these peptides elicit strong CD4+ and CD8+ antitumor responses. The peptide vaccines being considered for GBM are rindopepimut, IMA950, and IDH1 [89,111]. In a recent phase-II clinical trial, the administration of bevacizumab plus concomitant rindopepimut for rGBM showed a significant benefit both in terms of OS and PFS [112]. However, when administered in combination with TMZ in a randomized, double-blind, placebo-controlled phase-III clinical trial, it failed to show significant improvement in the 745 enrolled patients with pGBM [113]. Additionally, IMA950 failed to deliver robust results for the treatment of rGBM, with preclinical and clinical trials showing no benefit in patients treated with this peptide vaccine compared with the control cohort [89,114]. IDH1 mutations, expressed in more than 70% of rGBMs, are currently under investigation for a possible vaccine formulation, but despite some phase-I clinical trials being completed, relevant data are yet to be disclosed [89,115,116]. Unfortunately, some critical issues need to be addressed: firstly, GBM typically presents a scarce number of mutations, which reduces the number of potential TSA targets. Furthermore, it appears that single-antigen vaccines are insufficient to induce a durable antitumor response, an obstacle probably associated with the molecular heterogeneity of this peculiar malignancy, which ultimately leads to antigen escape [89,110].

### 5.4. Dendritic Cell Vaccines

This strategy aims to boost the immune system, driving it into a more efficient defensive state against cancer cells. For this purpose, dendritic cell (DC) vaccines condition T helper cells into activating the cytotoxic arm of the host immune system [117]. Currently, both single-specific- and multiple-antigen vaccines are under evaluation in clinical trials, with the latter strategy exhibiting better results due to the enhanced ability to limit antigen escape [89]. A phase-I/II clinical trial enrolling 22 patients with rGBM showed that the administration of a-type 1 polarized DCs loaded with EphA2, IL13Ra2, tyrosine (Y) lysine (K) and leucine (L) 40 kDA (YKL-40), and glycoprotein 100 kDA (gp100), and combined with polyinosinic–polycytidylic acid stabilized with polylysine and carboxymethylcellulose (poly-ICLC), led to a PFS of at least 12 months in nine patients, even showing complete remission in one subject [118]. Although a recent vaccine (DCVax-L) was obtained from a tumor lysate, DC vaccines are usually derived from ex vivo cells. Notwithstanding the promising results achieved in phase-I and -II clinical trials, with some patients exceeding 10 years of survival, consistent limitations are associated with the necessity to obtain tumor samples to customize the vaccine, thereby excluding patients with inoperable disease [87,119]. Notably, this technique may expose patients to autoimmune reactions, thus requiring further investigation [89]. Despite ongoing preclinical studies, speculation indicates that the DC vaccine followed by chemotherapy could substantially increase survival in patients with invasive gliomas [120].

### 5.5. Oncolytic Viruses

Although originally designed to increase tumor susceptibility to chemotherapy, oncolytic virus-based pharmacotherapy has recently been included in the immunotherapy panorama as an independent field [87]. The primary objective of oncolytic viruses is to induce cancer cell death, as well as to dismantle the tumor microenvironment concurrently activating inflammatory and immune mechanisms against the tumor itself [121]. Delivery options include intra or postoperative administration, and a wide range of viral species have been identified as possible vectors, including adenovirus, measles virus, retrovirus, poliovirus, vaccinia virus, and herpes simplex virus [89]. Promising results have been reported in a phase-I clinical trial enrolling 37 patients with rGBM, where the administration of the adenovirus DNX-2401 (NCT00805376) via single intratumoral injection was associated with a survival rate of at least 3 years in 20% of patients, with three of them demonstrating more than 3 years of PFS and a 95% tumor volume reduction [122]. This was the first study to show the potential benefits of oncolytic virus therapy for rGBM, and more results are expected to be reported in the upcoming months [89]. A recent comprehensive analysis of the effects of such a novel virotherapeutic strategy in progressive GBM demonstrated a positive impact on patients’ survival, with 2- and 3-year OS of 15% and 9%, compared with the 12% and 6% of non-virotherapy clinical trials conducted to date, respectively [123].

## 6. Current Guidelines for the Treatment of rGBM

For the purpose of this section, we reviewed four recent publications that we considered pivotal in the field of neuro-oncology, given their fundamental contribution to the understanding and management of progressive GBM [124,125,126,127]. In the pursuit to portray the most crucial and factual evidence regarding the multimodal management of rGBM, we hereby summarize the salient findings reported by the Congress of Neurological Surgeons (CNS) and EANO guidelines of the last two years.

### 6.1. CNS Guidelines on the Role of Radiation Therapy in rGBM

As with all of the systematic reviews considered in this section, the CNS evidence-based guidelines stem from an experienced and multidisciplinary commission of neurosurgeons, neuro-oncologists, and, in this case, specifically neuroradiologists, who gathered and reviewed the methodology and findings included in the previously published guidelines of 2014 [128], integrating additional recent research having potential relevance regarding re-irradiation for progressive GBM. After retrieving 311 publications from electronic databases (MEDLINE, Cochrane Database of Systematic Reviews, and Embase), the authors included in their evaluation nine papers that fulfilled the inclusion criteria. No prospective RCTs testing the role of radiation therapy with regard to OS and PFS were found. All the studies included in this review yielded a Level III recommendation or lower, as defined by Olson [129], therefore failing to achieve higher classes of evidence compared with previous guidelines. All nine studies proved that re-irradiation could be safely adopted in adult and elderly rGBM patients, suggesting favorable results in terms of tumor control, PFS, and OS, as well as functional outcomes and reduction in steroid dependence [130,131,132,133,134,135,136,137,138]. The authors also concluded that current evidence does not suggest any correlation between MGMT promoter methylation or positivity and other molecular markers, including IDH1, and survival in progressive GBM managed with re-irradiation. Additionally, no superiority data favored different RT modalities, including fractionated and non-fractionated SRS, and intensity-modulated RT, differing from the results we reported above.

### 6.2. CNS Guidelines on the Role of Cytoreductive Surgery in rGBM

Following the previously described methodology [129], the panel of experts searched the recent literature for publications exploring the role of repeat surgery in adult patients presenting with progressive GBM. Two prospective and two retrospective studies met all of the inclusion criteria and were, therefore, included in the systematic review. A total of 168 patients underwent reoperation for rGBM from the two prospective studies. Suchorska et al. [139] concluded that a second cytoreductive surgery improved the quality of life and survival when total resection of gadolinium-enhanced tumors could be safely achieved. Conversely, Yong et al. [140] found that post-resective functional improvement and OS were strongly dependent on residual tumor volume following reoperation, with tumors located in eloquent areas and large preoperative tumor volumes being associated with significantly poorer outcomes, possibly due to the inability to perform gross-total resections in such cases. As for the retrospective studies, promising results were found in reoperation sub-populations compared with those who did not receive surgical treatment or only underwent tumor biopsy in terms of survival benefit. Despite the significant improvement in OS (622.5 vs. 221 days; *p* = 0.041) in patients undergoing full resection described by Hager et al. [141], older age and lower KPS seemed to be related to higher risks of systemic complications from re-intervention [142].

### 6.3. CNS Guidelines on the Role of Cytotoxic Therapies in rGBM (TMZ Monotherapy)

The aim of the 2022 CNS guidelines on cytotoxic therapy in the management of rGBM was to assess the role of TMZ alone or in combination with other cytotoxic agents, including nitrosourea, cisplatin, and tamoxifen, and the use of tumor-treating fields (TTF) in adult patients with progressive disease. A broad analysis of the literature specifically focused on cytotoxic therapies yielded a total of forty-three publications adhering to all inclusion criteria. Among the collected citations, six studies focused on the role of TMZ administered at doses different from Stupp’s protocol [2], and an additional six publications evaluated TMZ in combination with other cytotoxic agents [143,144,145,146,147,148,149,150,151,152,153]. With regard to the metronomic administration of TMZ (25–50 mg/m^2^ daily), the authors concluded that such protocols also show discrete efficacy in terms of OS and PFS in the case of patients previously treated with bevacizumab. Nonetheless, due to the paucity of subjects enrolled in these studies, further evaluations are foreseen to achieve higher levels of recommendation. As for the administration of dose-dense TMZ (dd-TMZ) in progressive GBM, two phase-II trials investigated the efficacy and safety of 150 mg/m^2^ of dd-TMZ administered on days 1–7 and days 15–21 of a 28-day cycle for a total of 12 cycles after recurrence and of 75–100 mg/m^2^/day dd-TMZ for 21 days every 28 days. In both cases, the primary outcomes were PFS at 6 months and, although the two administration regimens demonstrated a good safety profile, neither of them succeeded in achieving said results.

### 6.4. CNS Guidelines on the Role of Cytotoxic Therapies in rGBM (TMZ Combinations)

The authors further proceeded to examine the role of combinations of TMZ with additional cytotoxic agents, such as nimustine, cisplatin, irinotecan, tamoxifen, fotemustine, and lomustine, considered in different publications [149,151,154]. Unfortunately, due to the lack of power of these studies, with the majority being single-armed or retrospective in nature, providing only Class III evidence, none of the examined publications showed robust evidence favoring alternative dosing of TMZ compared with conventional TMZ alone or in combination with other cytotoxic agents. Promising results have been reported favoring the administration of fotemustine in patients with progressive disease, showing positive outcomes and safety profiles, especially in the elderly population with MGMT-positive rGBM relapsed after TMZ schedules [155,156,157,158,159]. Commonly evaluated administration protocols included 120 mg/m^2^ every two weeks for up to 1 year and 70–100 mg/m^2^ weekly for 3 weeks and then every 3 weeks as maintenance therapy, with the most frequent adverse events represented by grade-3 and -4 thrombocytopenia and grade-3 and -4 neutropenia.

### 6.5. CNS Guidelines on the Role of Cytotoxic Therapies in rGBM (TTF)

Lastly, an investigation of the therapeutical role and tolerability of TTF for the management of progressive GBM was performed. Such cytotoxic therapy is grounded in the ability of non-invasive, regional low-intensity (1–3 V/cm), intermediate-frequency (100–300 kHz) alternating electric fields to cause cell death, probably secondary to the misalignment of microtubule subunits in the mitotic spindle during the metaphase [160,161]. Five studies were evaluated for this purpose, yielding Class II evidence supporting the use of this innovative cytotoxic therapy that provides similar survival rates with decreased toxicity when compared with conventional chemotherapy [162,163,164,165]. The most promising results were achieved by the combination of TTF with TMZ, bevacizumab, and irinotecan in a cohort of 30 patients with refractory GBM, and by the combination of pulse bevacizumab + TTF. Nonetheless, due to the paucity of controlled double-armed trials and the small populations investigated to date, future studies on the efficacy of TTF alone or in combination with chemotherapy in the management of rGBM are warranted.

### 6.6. EANO Guidelines for the Treatment of rGBM

In their recently published article, the European task force aimed to formulate evidence-based recommendations based on the latest results derived from clinical trials, as well as other relevant studies, and define the role of different treatment strategies for the management of diffuse gliomas [124]. For the purpose of this review, we only focused on the data concerning rGBM. The authors suggested a relative beneficial impact of SRT and radiosurgery on the management of progressive disease, rather than whole-brain radiotherapy, especially for their ability to spare non-malignant brain tissue usually already markedly altered in such populations due to the repetitive cytoreductive surgeries and extensive tumor infiltration. Suggestions also include the possibility of re-irradiation for patients presenting with disease relapse following standard protocols after ~12 months, but the class of evidence does not exceed III, given the absence of RCTs conducted to date. For instance, controlled prospective double-armed studies evaluating the tolerability, safety, and efficacy profiles of these approaches compared with standard RT are warranted [166,167]. As for the pharmacological recommendations, the results do not significantly differ from those reported in the previous sections. As alkylating cytotoxic agents should be considered for subjects presenting with progressive GBM who did not receive previous adjuvant chemotherapy, the current evidence fails to demonstrate the overall efficacy of this strategy. Additionally, similar to the abovementioned results, TMZ and nitrosoureas present comparable efficacy profiles in the treatment of rGBM [168,169], and the combination of TMZ with bevacizumab or nivolumab does not show favorable outcomes in terms of OS and PFS in the setting of contrast-enhancing progressive IDH-positive GBM without 1p/19q codeletion [170,171].

## 7. Summary and Future Directions

As outlined in this review, different treatment strategies are currently under investigation to improve OS and quality of life in patients affected by progressive GBM. Innovative approaches include immunotherapy and targeted molecular pharmacotherapy, demonstrating promising Class II and III results. For instance, based on the molecular and genetic profiling of the malignancy, it is possible to inhibit tumor-specific targets, as in the case of bevacizumab or osimertinib, or multiple kinase receptors, as demonstrated with regorafenib. These approaches have reached the most solid results so far, with significant improvements in terms of OS and PFS. Furthermore, despite the immunological privilege that characterizes the central nervous system, different authors have shown favorable outcomes with the use of adoptive T-cell and CAR-T strategies, which nonetheless need to be investigated in larger randomized cohorts. It must be noted that the distinctive antigenic profile of rGBM and the technical difficulties associated with its sampling hinder a more rapid development of engineered drugs and their testing in RCTs when compared with pGBM. It is, therefore, vital to include patients with rGBM in clinical studies to assess the role of newly developed agents also in this minority, and a more tailored therapeutical approach for patients presenting with progressive GBM based on the lesion’s footprints is foreseen.

## Figures and Tables

**Table 1:** Summary of clinical trials currently ongoing for investigating pharmacological agents for the management of rGBM.

**No. of clinical trials**	103
**Phase**	*Phase 1*: 52 (50.49%)
	*Phase 2*: 46 (44.66%)
	*Phase 3*: 5 (4.85%)
**No. of arms**	*1*: 56 (54.37%)
	*2*: 32 (31.07%)
	*>2*: 15 (14.56%)
**No. of enrolled patients**	*<50*: 66 (64.08%)
	*50–100*: 24 (23.3%)
	*100–200*: 10 (9.71%)
	*200–500*: 2 (1.94%)
	*>500*: 1 (0.97%)
**No. of systemic therapies as a therapeutical investigation**	*1*: 50 (48.54%)
	*2:* 41 (39.81%)
	*3*: 9 (8.74%)
	*>3*: 3 (2.91%)
**Combinations**	*Trials including radiotherapy*: 21 (20.39%)
	*Trials including surgery*: 32 (31.08%)
	*Trials including radiotherapy and surgery*: 7 (6.8%)
**Treatment allocation**	*Randomized*: 22 (21.36%)
	*Non-randomized*: 24 (23.3%)
	*n/a*: 57 (55.34%)
**Masking**	*None*: 95 (92.23%)
	*Single*: 1 (0.97%)
	*Double*: 4 (3.88%)
	*Other*: 3 (2.91%)
**Interventional model**	*Parallel assignment*: 26 (25.24%)
	*Sequential assignment*: 20 (19.42%)
	*Single-group assignment*: 56 (54.37%)
	*Crossover assignment*: 1 (0.97%)
**Country**	*USA*: 61 (59.22%)
	*International*: 14 (13.59)
	*China*: 12 (11.65%)
	*Norway*: 3 (2.91%)
	*Germany*: 2 (1.94%)
	*Others*: 11 (10.68%)
**Estimated date of completion**	*2022–2025*: 90 (87.38%)
	*2026–2030*: 12 (11.65%)
	*Beyond 2030*: 1 (0.97%)
**Most represented primary endpoints**	*Treatment-related adverse effects*: 34 (33.01%)
	*PFS*: 27 (26,21%)
	*Dose-limiting toxicity*: 26 (25.24%) *OS*: 22 (21.36%)

**Table 2:** Most represented drugs in clinical trials.

Drug Group	Agent	Trial Phase	Effect
Alkylating agents	TMZ (Temozolomide)	I: 7, II: 8, III: 2	DNA-alkylating agent, whose effect mostly occurs at the N7 or O6 positions of guanine residues. DNA modification may induce the death of tumoral cells. The drug efficacy might be hindered by the enzyme MGMT.
	Lomustine	I: 2, II: 3, III: 1	Bifunctional alkylating agent (effect both on DNA and RNA). In DNA, it creates interstrand cross-links. Owing to its ability to carmaboylate on aminoacidic residues of proteins, its effect might be further increased by inhibiting several key enzymatic processes.
	VAL-083	I: 0, II: 0, III: 1	Bi-functional alkylating agent—its effects are expressed through cross-linking with an epoxide group along all phases of the cell cycle.
Anti-angiogenic	Bevacizumab	I: 1, II: 7, III: 2	Inhibitor of VEGF-A, causing the inhibition of angiogenesis
Immune checkpoint inhibitors	Nivolumab	I: 4 II: 3 III: 0	Preventing PD-L1-induced T-cell inactivation by binding PD-1 to its extracellular domain
	Ipilimumab	I: 4, II: 1, III: 0	Avoiding T-cell inactivation by binding CTLA-4 receptors
	Pembrolizumab	I: 1, II: 3, III: 0	Preventing PD-L1-induced T-cell inactivation by binding PD-1 on its extracellular domain
PARP inhibitor	Niraparib	I: 1, II: 2, III: 0	Preventing tumor cells’ DNA reparation, and consequently inducing tumor cell death by inhibiting PARP1/2
Adoptive T-cell therapy	CAR-T B7-H3	I: 3, II: 1, III: 0	Allows the T-cells to recognize B7-H3 in order to increase the immunological response
Topoisomerase inhibitor	Irinotecan	I: 2, II: 1, III: 0	Traps a subset of topoisomerase-1-DNA, avoiding tumor cells’ DNA replication
Autologous dendritic cell	ADCTA	I: 0, II: 0, III: 1	Elicitation of antigen-specific, CD4/CD8 cytotoxic T-cells’ responses and induction of IFN-γ secretion
FASN inhibitor	ASC40	I: 0, II: 0, III: 1	Induction of the depletion of long-chain fatty acids, consequently leading to cell death by inhibiting FASN, which is preferentially expressed in malignant tissues
PI3K/mTOR inhibitor	Paxalisib	I: 0, II: 0, III: 1	Inhibition of cell growth/survival by specifically inhibiting PI3K in the PI3K/AKT kinase signaling pathway
VEGFR2-TIE2 tyrosine kinase inhibitor	Regorafenib	I: 0, II: 0, III: 1	Anti-angiogenic activity by inhibiting VEGFR2-TIE2 tyrosine kinase
JAK1/3 inhibitor	Tofacitinib	I: 0, II: 0, III: 1	Influence on DNA transcription by inhibiting JAK1/JAK3 and interfering with the JAK-STAT pathway

## Data Availability

Not applicable.
